# Direct Depth- and Lateral- Imaging of Nanoscale Magnets Generated by Ion Impact

**DOI:** 10.1038/srep16786

**Published:** 2015-11-20

**Authors:** Falk Röder, Gregor Hlawacek, Sebastian Wintz, René Hübner, Lothar Bischoff, Hannes Lichte, Kay Potzger, Jürgen Lindner, Jürgen Fassbender, Rantej Bali

**Affiliations:** 1 Triebenberg Labor, Institut für Strukturphysik, Technische Universität Dresden, D-01062 Dresden, Germany; 2 Helmholtz-Zentrum Dresden-Rossendorf, Institut für Ionenstrahlphysik und Materialforschung, Bautzner Landstraße 400, D-01328 Dresden, Germany; 3 Institut für Festkörperphysik, Technische Universität Dresden, Helmholtzstr. 10, D-01069 Dresden, Germany

## Abstract

Nanomagnets form the building blocks for a variety of spin-transport, spin-wave and data storage devices. In this work we generated nanoscale magnets by exploiting the phenomenon of disorder-induced ferromagnetism; disorder was induced locally on a chemically ordered, initially non-ferromagnetic, Fe_60_Al_40_ precursor film using 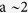 nm diameter beam of Ne^+^ ions at 25 keV energy. The beam of energetic ions randomized the atomic arrangement locally, leading to the formation of ferromagnetism in the ion-affected regime. The interaction of a penetrating ion with host atoms is known to be spatially inhomogeneous, raising questions on the magnetic homogeneity of nanostructures caused by ion-induced collision cascades. Direct holographic observations of the flux-lines emergent from the disorder-induced magnetic nanostructures were made in order to measure the depth- and lateral- magnetization variation at ferromagnetic/non-ferromagnetic interfaces. Our results suggest that high-resolution nanomagnets of practically any desired 2-dimensional geometry can be directly written onto selected alloy thin films using a nano-focussed ion-beam stylus, thus enabling the rapid prototyping and testing of novel magnetization configurations for their magneto-coupling and spin-wave properties.

Magnetic nanostructures are of critical importance for spin-transport devices^1^ as well as in devices exploiting magnetically coupled structures for spin-wave manipulation (Magnonics)^2^,^3^. Significant efforts are focused on fabricating high density arrays of magnetic nanostructures, where each nanostructure can act as a magneto-logic element or as a binary data bit^4^,^5^.

Achieving the varied geometries and small sizes of magnetic objects, necessary for these applications, is challenging. For instance, spin-transport applications require magnetic nanostructures separated by non-magnetic conducting barriers. Such structures are typically hetero-structures containing several interfaces that result in spin-scattering losses. Moreover, these devices can require a number of patterning and electrical contacting steps. Similarly, magnonic devices may require wave-guides of various shapes and defined magnetization configurations to enable spin-wave propagation^6^.

A solution to patterning magnetic nanostructures of desired geometries is to write magnetic objects directly onto a non-magnetic precursor by the use of an energetic ion beam. It has been shown that the saturation magnetization, *M*_s_, of certain alloys can be drastically increased due to the chemical disordering^7^. Chemical disordering can be induced locally by impinging ions, thereby producing confined magnetic structures^8^,^9^. The resulting magnetic structures are embedded within an electrically conducting non-magnetic matrix, avoiding the structural interfaces that act as spin-scattering sites present in hetero-structures.

Ion-irradiation can modify a variety of magnetic properties in alloys, including the magnetization easy axes, magnetic anisotropy, coercive fields and ferromagnetic resonances^10^,^11^. Most of these cases involve the irradiation of a magnetic precursors, and while the above properties are changed significantly, the *M*_s_ itself remains fixed or is reduced due to ion-induced damage of the crystal structure or alloying with the ion-species. The converse is true in certain cases, whereby magnetization is induced in regions that interact with the ions. A variety of mechanisms can be exploited such as ion-induced magnetization due to a fcc-bcc phase transitions in ultrathin Fe films^12^, local reduction of metal oxide with H^+^^13^, or by chemical disordering in certain paramagnetic B2-ordered alloys, locally transforming them to a ferromagnetic A2 phase^8^.

Here we focus on ion-induced magnetization due to chemical disordering, taking Fe_60_Al_40_ thin films as our model system. This is a powerful way to directly write magnetic nanostructures using ions, as it is a physical process that involves the displacement of only a few atoms within the lattice to generate large magnetization, while preserving the flat topography of the original film. Alloys possessing disorder-induced ferromagnetism phenomena usually consist of atoms of magnetic and non-magnetic species, and magnetic phase transitions can be triggered by chemical disordering. That is, a chemically ordered thin film acts as a template onto which disordered regions can be printed using localized exposure to an ion beam. Ions penetrating through a chemically ordered structure form collision cascades, where disorder is formed locally, and ferromagnetism is generated due to an increase in the number of magnetic moment carrying nearest-neighbours^14^,^15^,^16^

To produce magnetic nanostructures it is necessary to confine the volume over which the ions interact with the host atoms. One approach is to irradiate through lithographically patterned shadow masks^8^,^12^,^13^,^17^, however the patterning resolution is greatly influenced by the lithography process, rather than by the interaction volume. Further confinement of ion-magnetized regions can be achieved by a narrow ion-beam, where the beam diameter is ideally much smaller than the lateral dimensions of the interaction volume. Here the focused beam of ions could act as a stylus for direct magnetic writing.

State-of-the-art instruments such as the He-ion Microscope (HIM) can focus ions down to 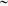
0.5 nm, while scanning the beam to form the desired magnetic patterns^18^. The technique is based on a gas field ion source. Three atoms situated on the apex of sharp metal tip are used to create a beam of field ionized noble gas atoms such as He^+^ or Ne^+^. For high resolution work the beam tails are reduced to a minimum by using a small aperture and placing the beam crossover at a large distance from the aperture. This ion-optics design ensures that ions originating from a single ionization site only (*i.e.*, only one of the three apex atoms) can reach the sample, resulting in a narrow beam of 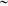
2 nm diameter in case of Ne^+^ ions. It is difficult to estimate the profile for such narrow beam widths^19^. Nevertheless it has been shown that the 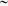
2 nm beam diameter for ions at typical energies of 20 to 30 keV is far narrower than the lateral dimensions over which magnetization is expected to be induced^8^. Here we deploy the nano-focussed ion beam of a HIM to generate confined magnetic stripes on a template of chemically ordered Fe_60_Al_40_ (Fig. 1).

The distribution of atomic displacements caused by impinging ions tends to be spatially inhomogeneous. An ion penetrating a crystalline material undergoes deceleration due to the crystal potential and collisions with the nuclei, until the ion is stopped completely at a certain depth. Nuclear collisions can cause atoms to be knocked out from their lattice sites, which results in recoiling ions and atoms in arbitrary directions. The collision processes are inhomogeneous over the depth- and lateral- directions, and modifications to magnetic properties resulting from the atomic displacements, such as the strongly increased magnetization, may consequently also follow an inhomogeneous spatial distribution. Spatial inhomogeneity limits the resolution of the patterned magnetic objects and determines their functionality as device elements. The spatially resolved effect of low dose damage cascades on functional properties is typically challenging to visualize and with few exceptions^20^, one has to trust simulations of the ion trajectories. In this work we generate magnetic objects using ion-irradiation and directly observe flux lines of the magnetized regions at the nanoscale. Estimations of the homogeneity and sharpness of ion-induced magnetic patterns have been achieved *via* direct observations.

Observations of the depth- and lateral- distribution of the flux lines of nanostructured objects were performed using Transmission Electron Microscopy (TEM) combined with electron holography. Off-axis electron holography was employed to observe and quantify the depth- and lateral- distribution of the magnetic flux density of ion-magnetized structures in Fe_60_Al_40_. In case of continuous thin films which have their magnetization, *M*, confined to the film plane, the magnetic flux density within the film is given by *μ*_0_*M*. Therefore *μ*_0_*M* can be measured across the film thickness for the ion-magnetized films discussed here. The value of *μ*_0_*M* was observed to strongly vary depending on the ion-penetration depth.

Lateral magnetic patterning introduces boundaries within the sample where the continuity of the magnetization is broken and stray flux lines leak from the edges of the magnetic structures. Electron holography was employed to laterally trace the stray field in order to draw conclusions on the magnetization variation around the magnetic edge regions. It is shown that the stray flux density in the vicinity of edges of magnetic structures depends sensitively on the nanoscale sharpness of the edge.

Electron holography revealed the depth-distribution of *μ*_0_*M* induced by ions of different energies penetrating Fe_60_Al_40_ thin films. Furthermore, the lateral-distribution of *μ*_0_*M* for bar magnet-like objects separated by 100 nm wide spacings is estimated. The direct confirmation of well-defined magnetic structures patterned using an ion-beam shows that ion-induced ferromagnetism is a powerful approach to pattern homogeneous and defined nanomagnets for a vast variety of applications.

## Results

Before studying magnetically patterned samples, we observed the depth variation of the magnetic flux distribution in 40 nm thick, B2 ordered Fe_60_Al_40_ films that were uniformly irradiated with Ne^+^ ions at various ion-energies. In addition we also observed the magnetic flux-density within an unirradiated B2 ordered film as control sample. These measurements were necessary to show that the depth-profile of the induced magnetization is a function of the ion-energy, and above a certain energy the films can indeed be homogeneously magnetized using energetic ions, which can be important for patterning functional magnetic objects.

**Figure 2:**
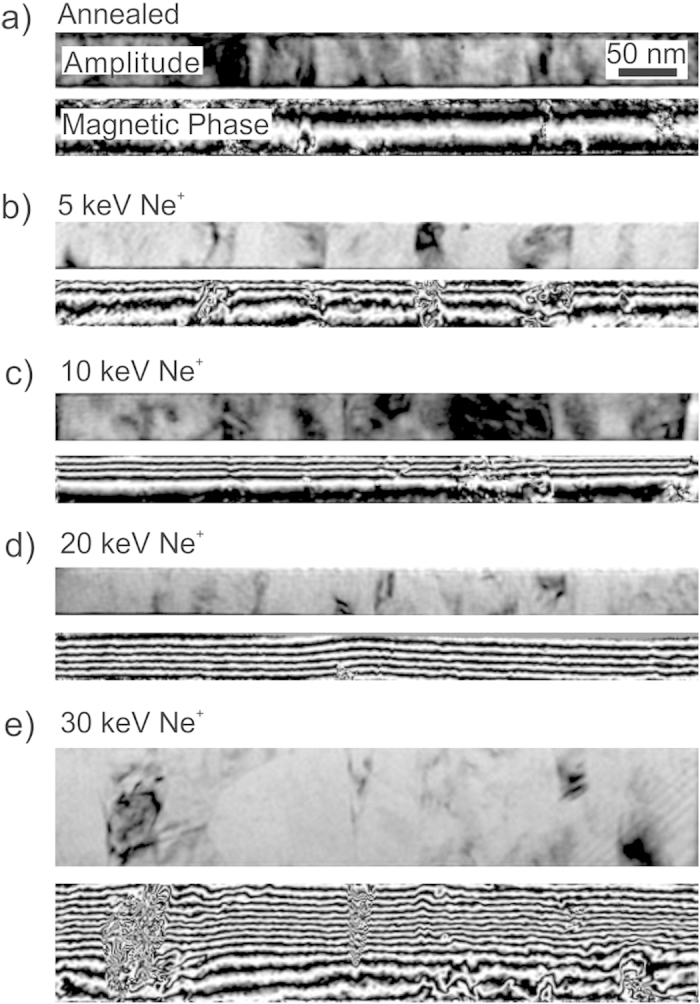
Magnetic flux lines induced by ion-irradiation of Fe_60_Al_40_. Amplitude images show structural information whereas the corresponding magnetic phase (φ_mag_) images show the distribution of magnetic flux lines. The Fe_60_Al_40_ thin films were annealed at 773 K in vacuum prior to ion irradiation, and (**a**) shows the amplitude and magnetic phase for the annealed film. The annealed films were irradiated with Ne^+^ ions at energies of (**b)** 5, (**c)** 10, (**d)** 20, and (**e)** 30 keV. The Ne^+^ fluence is 6 × 10^14^ ions cm^−2^. The films in (a–d) are 40 nm thick, and 100 nm thick in (d). Magnetic flux lines have been visualized by plotting the spatial variation of the cosine of the magnetic phase shift, cos(φ_mag_). To enhance contrast and compensate for different thicknesses of the cross-sections, φ_mag_ has been amplified by a factor *i.e.*, the plots show (a) cos(5.2φ_mag_) (b) cos(20.7φ_mag_) (c) cos(6φ_mag_) (d) cos(12φ_mag_) and cos(22.2φ_mag_).

The depth distribution of the ferromagnetic A2 phase formed due to atomic displacements depends on the ion-energy; the depth of the magnetized region (or the A2/B2 phase boundary) with respect to the film surface can be expected to increase with increasing ion-energy. Figure 2 shows holographic images for 40 nm thick Fe_60_Al_40_ films that were initially in the B2 phase, and subsequently irradiated with 5, 10 and 20 keV Ne^+^ ions with a fluence of 6 × 10^14^ ions cm^−2^; as well as a 100 nm thick Fe_60_Al_40_ film irradiated with 30 keV Ne^+^ ions with the same fluence.

The fluence is rather low such that ion-induced variations of the grain structure and morphology are negligible^8^. On the other hand, ion-irradiation causes significant changes to the chemical ordering. Binary collision approximation simulations show that each ion is expected to displace 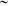
100 atoms at 5 keV or 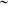
500 atoms at 30 keV, forming dense networks of collision cascades^21^. The fluence has been selected based on simulations, such that 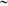
1 displacement per atom occurs at the peak for the above ion-energies. The estimated sputtering is approximately 3 atoms per ion, corresponding to 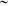
1 atomic plane being removed for the above fluence. Furthermore, it has been shown experimentally that for a Ne^+^ fluence of 6 × 10^14^ ions cm^−2^ the maximum *M*_s_ is reached within the ion-affected regions, for all of the above ion-energies^8^. The previous work was based on measurements of *M* integrated over the whole sample volume, whereas here we perform local observations of the variation of 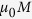 with film depth.

A convenient way to show magnetic phase images is by plotting the cosine of the magnetic phase shift, φ_mag_. To help isolate the contribution of φ_mag_ it is necessary to apply magnetic fields *insitu*, which has been done using the magnetic field of the objective lens^22^. To increase the contrast and to compare samples with different thicknesses, φ_mag_ is usually multiplied by an amplification factor. Plotting cos(amplified φ_mag_) directly visualizes the projected magnetic flux density within the sample plane. For each sample shown in Fig. 2, there are two panels corresponding to the amplitude and magnetic phase image, respectively. The film structure can be seen in the amplitude images, where grains of approximately 50 nm width are observed. The corresponding magnetic phase images show the distribution of the projected magnetic flux lines. The images were captured in remanence after having applied a large saturating in-plane magnetic field of approximately 1.0 T. We may therefore assume that the magnetization estimated from holography is equal to the saturation magnetization (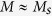). Figure 2a shows the flux lines in the B2 phase, prior to any ion-irradiation. This control sample shows a residual flux-density, likely to have been induced by atomic displacements during sample preparation (see methods). Upon irradiation with Ne^+^-ions at 5 keV (Fig. 2b), the flux-density is seen to increase at the surface region, and decay with film depth. Disturbances of the flux lines seen in the phase images correspond to structural features seen in the amplitude image where the phase estimation is less reliable. Aside from disturbances, coherent flux lines were observed in the vicinity of the film surface, while inside the film the flux line density decreases rapidly with increasing distance from the surface.

As the ion-energy is increased to 10 keV (Fig. 2c), the region with increased flux-density penetrates to approximately 20 nm film depth. Increasing the ion-energy further to 20 keV (Fig. 2d), results in a largely homogeneous flux density throughout the film. To observe the effect of Ne^+^-ions at a higher energy, a 100 nm thick film was irradiated with 30 keV Ne^+^ ions (Fig. 2e). The flux-line distribution shows a partially magnetized film, consistent with cases where the ions possess insufficient energy to fully penetrate the B2 film. Thus in all irradiated films, there occurred a region of increased flux density, with the depth of the magnetized region increasing with increasing ion-energy. The maximum magnetic flux density of the magnetized region was estimated to be 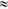
1.0 T (see Supplementary Information), consistent with previous reports^8^. The above trend in the flux density distribution as a function of ion-energy will be analyzed in further detail in the discussion section.

Next, we investigated a magnetically structured sample in an attempt to observe the lateral distribution of the flux lines. A separate sample was irradiated using a HIM with a nano-focussed Ne^+^ beam at 25 keV and a fluence of 6 ions nm^−2^ (the same as for the continuous films). A 10 μm beam limiting aperture and a very short dwell time of 0.1 μs have been used to write the pattern at a current of 0.8 pA. To achieve the desired fluence while reducing inhomogeneities related to the low beam current each pattern was scanned several times. The resolution of the patterned structures is limited by the lateral scattering of ions.

**Figure 3:**
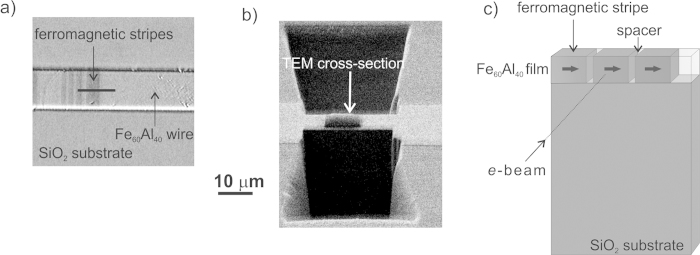
Magnetic stripe pattern and cross-sectioning. (**a**) Magnetic contrast image obtained using Kerr effect of a 10 *μ*m wide Fe_60_Al_40_ wire onto which magnetic stripes have been patterned using a nano-focussed Ne^+^ ion beam. The patterned regions appear as dark contrast, and consist of bunches of 5 stripes separated by 100 nm wide spacings. A region containing 500 nm wide stripes was selected for cross-sectioning, indicated by the solid black line. (**b**) Scanning Electron Microscopy image of the TEM cross-section prior to extraction. (**c**) Schematic of the extracted cross-section. The block arrows indicate the direction of magnetization prior to holographic imaging.

A magnetic stripe-pattern was written directly onto a 10 *μ*m wide wire using the nano-focussed beam. The ion-beam consisted of Ne^+^ ions at 25 keV *i.e.*, with energy sufficient to fully penetrate the 40 nm thick Fe_60_Al_40_ film. The 2-dimensional stripe pattern consisted of 500 nm wide irradiated regions, separated by 100 nm wide spacings. Magnetic contrast was observed using the Kerr effect in an optical microscope (Fig. 3a), where the magnetized regions show dark contrast. The resolution achievable in the optical microscope was insufficient to resolve the 100 nm wide separations between the magnetized stripes. To observe the magnetization distribution in the nominally 100 nm wide spacer regions, a cross-section was carved using a beam of Ga^+^ ions (Fig. 3b). The cross-section, containing a few patterned stripes was lifted out and fine polished (see Methods). A schematic of the cross-section is shown in Fig. 3c. Prior to imaging a saturating magnetic field was applied such that the magnetization within the stripes was aligned along the 500 nm wide stripe edges, as depicted by the block arrows in Fig. 3c.

**Figure 4:**
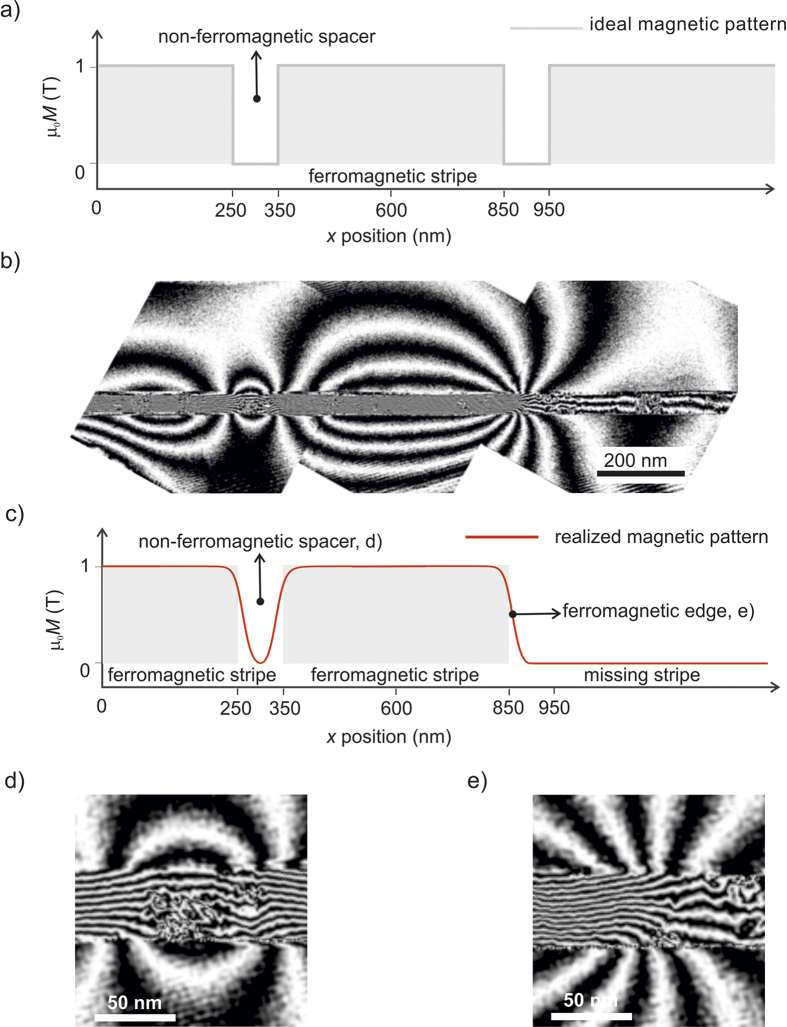
Magnetic patterning using ions. (**a**) Nominal schematic of the 500 nm wide ferromagnetic stripes separated by 100 nm wide non-ferromagnetic spacers, written using a nano-focussed Ne^+^ ion beam. (**b**) Holographic images of the flux lines of the patterned magnetic stripes. (**c**) Schematic of the experimentally realized ferromagnetic stripe pattern, showing the blurring of the edges due to lateral-ion scattering and the missing ferromagnetic stripe on the right. High magnification images of the flux density in selected regions *viz.* (**d**) the non-ferromagnetic spacer between two ferromagnetic stripes and (**e**) the ferromagnetic edge at the end (right) of the central ferromagnetic stripe.

The sample region investigated was expected to cover three ferromagnetic stripes, with one in the centre and two half-stripes on the left and right side respectively. The ferromagnetic stripes were to be separated by 100 nm wide non-ferromagnetic spacers. Figure 4a shows the model lateral profile of the intended magnetic structure, where the maximum magnetization, *M*, in the stripes corresponds to a magnetic flux density of 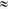
1 T. The holographically reconstructed flux line distribution, visualized here by plotting cos(20φ_mag_), is shown in Fig. 4b. The realized magnetic pattern deviates from the ideal pattern. A schematic of the realized pattern is shown in Fig. 4c. The two key differences between the nominal and the realized patterns are 1) the missing magnetic stripe on the right-side, and 2) the existence of regions with transient magnetization at the interfaces between the expected ferromagnetic and non-ferromagnetic regions.

The solid black line in Fig. 3a shows that the lamellar TEM cross-section may contain both patterned as well as unpatterned regions, with the edge of the patterned region lying approximately at the lamella center. The most likely reason for the “missing” ferromagnetic stripe seen in Fig. 4b is therefore that the region observed with holography consisted of with the magnetic edge and the surrounding unpatterned area on the right. This coincidental positioning of the magnetic pattern with respect to the region of interest provided an opportunity to observe the stray field lines around a ferromagnetic edge, free of interference from the stray-field lines from neighbouring ferromagnetic stripes.

The flux-line distribution of the non-ferromagnetic spacer region between the two well-formed stripes is shown in higher magnification in Fig. 4d. A higher magnification image of the region containing the ferromagnetic edge, formed between the central ferromagnetic stripe and the missing ferromagnetic stripe on the right, is shown in Fig. 4e. The ferromagnetic-edge region is free of structural defects and particularly useful for analyzing the effect of lateral ion-scattering, discussed later in the text (see amplitude image in the Supplementary Information).

From the magnetic phase image of Fig. 4b it can be seen that magnetic patterning of two ferromagnetic stripes was realized. This can be deduced from the coherent flux lines within the film in the region corresponding to the half-stripe on the left, and the full stripe in the center. Stray flux lines are observed emerging from the edges of the well-formed ferromagnetic stripes. The stray flux lines are formed due to magneto-static coupling between the magnetic poles of the stripes. Stray flux lines are seen to connect the north and south of the central stripe, as well as to connect two neighbouring stripes through the non-ferromagnetic spacer regions. Some flux lines are also formed at defect sites within the well-formed stripes, showing the existence of magnetic poles at these sites. The existence of defects of such narrow widths is indicative of the high resolution of magnetic patterning that can be realized using the phenomena of ion-induced ferromagnetism.

Figure 4d shows the non-ferromagnetic spacer region, where flux lines connect the two neighbouring ferromagnetic stripes causing inter-stripe magneto-static coupling. Flux lines within the spacer regions are found to be slightly incoherent, due to structural defects similar to those seen in the uniformly irradiated films of Fig. 2 (see amplitude image in Supplementary Information). Nevertheless, classical stray field lines expected to traverse between two bar magnets can be observed in the vicinity of the metallic film *viz.* in the non-ferromagnetic regions above and the substrate region below.

The region corresponding to the half-stripe on the right remained non-ferromagnetic, as observed by the significantly reduced density and the incoherence of the flux lines in this region. Furthermore stray flux-lines do not originate from this region, as is the case with the two ferromagnetic stripes. The reason for the nearly vanishing flux-density of this region is unclear; however it is likely that the ion-beam missed irradiating this particular region.

At the ferromagnetic-edge region shown in Fig. 4e, stray flux lines emerge from the magnetized region on the left, into the non-ferromagnetic part of the film on the right, and into the vacuum and substrate regions. Stray flux lines from the corners traverse towards the opposite end of the same ferromagnetic stripe, as expected of a classical bar magnet. These flux lines are not disturbed by any flux lines from neighbouring stripes. This fact in combination with high structural integrity of this region helps constructing a simple model for describing the experimental results (see Supplementary Information). The ferromagnetic-edge region will be used for estimating the effect of lateral-scattering on the induced magnetization. Before we obtain the analytical fitting of the flux lines at this ferromagnetic-edge, it is necessary to analyze the depth-variation of the flux density in continuous thin films.

## Discussion

We return to the continuous thin film samples depicted in Fig. 2, in order to quantify the observed depth distribution of the magnetic flux density and to attempt to fit the distribution with an empirical model reported previously^8^.

**Figure 5:**
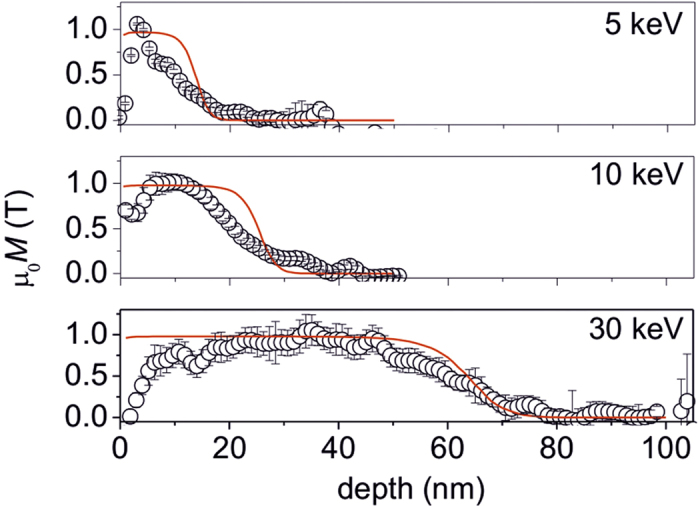
Depth penetration of ion-magnetized regions. Depth profiles of the flux density due to the magnetization, *M*, induced by Ne^+^ ions at 6 × 10^14^ ions cm^−2^ and given ion-energies. Red lines show calculations using an empirical model that considers the displacements undergone by atoms due to impinging ions. See Supplementary Information for details on error bar estimation.

The magnetic flux density plotted as a function of film depth after Ne^+^ irradiation at 5, 10 and 30 keV is shown in Fig. 5. The *μ*_0_*M* values shown on the vertical axis have been extracted from the gradient of the magnetic phase shift (see Methods), normalized by subtracting a background and dividing by the effective thickness of the sample in the measured region (see Methods and Supplementary Information). For the samples shown in Fig. 5, the ions did not possess sufficient energy to penetrate the full film. Beyond the ion-penetration depths for the given energies, *μ*_0_*M* decays and reaches a floor value. In Fig. 5, the *μ*_0_*M* floor has been assumed to be 0 T, because the magnetization of the non-irradiated material has been shown to be negligible^8^. Figure 5 does not show the depth variation of *μ*_0_*M* for the 20 keV irradiated sample. As shown in Fig. 2d, the films irradiated with 20 keV Ne^+^ ions show well-formed flux lines throughout the film thickness, showing that the ions induced magnetization homogeneously throughout the 40 nm thick Fe_60_Al_40_ film. The fact that the full volume of the film has been magnetized has also been confirmed by magnetometry^8^. However, a fully magnetized film lacks a reference non-magnetized region necessary for background correction and, precluding an estimation of local *μ*_0_*M*.

The depth variations of *μ*_0_*M* (Fig. 5) show that the effective thickness of the ion-magnetized region increases with increasing ion-energy. Within certain depth ranges *μ*_0_*M* decays from 1 T to 0. The *μ*_0_*M* decay commences at depths of 5, 15 and 45 nm, respectively, for 5, 10 and 30 keV ion-energy. Figure 5 also shows the predicted flux density distribution using the semi-empirical model from Bali *et al.*^8^ This model was proposed by measuring the integral magnetization of the ion-irradiated films and correlating these integral values to the depth variation of the atomic displacements, calculated using the binary collision approximation^21^.

The direct observations shown in Fig. 5 are in-line with the predictions of ref. 8. In particular the depths at which the flux density nearly vanishes show a near-exact match. The experimentally observed depth variation of the magnetic flux density also shows some deviations from the prediction. Irrespective of the ion-energy, *μ*_0_*M* decays sharply with decreasing depth, from 5 nm up to the surface. A thin surface oxide layer of <5 nm thickness can be expected to form and contribute to the *μ*_0_*M* decay. Moreover the interpretation of the behavior of the flux density in the vicinity of the surface is difficult, because of alignment and defocus mismatches as well as phase-unwrapping artifacts emerging through both limited fringe sampling and reduced signal-to-noise ratio (see Supplementary Information for discussion on noise).

The decay of *μ*_0_*M* around depths of 15 and 20 nm for the 5 and 10 keV Ne^+^ ions respectively appears to possess a shallower slope than that of the prediction (Fig. 5). In the case of the 100 nm thick film irradiated with 30 keV Ne^+^ ions, the transient region around a depth of 60 nm agrees well with the prediction. However for this film irradiated with 30 keV Ne^+^ ions, a gradual decrease of *μ*_0_*M* is observed while approaching the surface from a depth of 20 nm. A number of assumptions are made in the binary collision approximation calculations^21^, which may lead to the experimentally observed deviations from the predictions. In particular, the positions of the atoms are assigned to be at random, essentially simulating an amorphous material and neglecting the effect of ions passing through the atomic spacings of the crystallites without any collisions, known as channeling. The polycrystalline Fe_60_Al_40_ films studied here possess grain sizes of 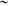
40 nm, and channeling may occur in grains with certain favorable crystallographic orientations. This implies that the semi-empirical calculations may overestimate the ion-induced magnetization in certain grains. The differences may arise due to the fact that the calculations are based on integral measurements whereas TEM focuses on a limited number of grains. Studies on epitaxial Fe_60_Al_40_ films would help improve our understanding of channeling effects, but are beyond the scope of this work.

Despite the assumptions in the modelling, the depth at which the ion-induced *μ*_0_*M* vanishes *viz.* 20, 30 and 70 nm for 5, 10 and 30 keV ion-energies respectively, is predicted with reasonable accuracy by the empirical model. This shows that ion-energy can be used as a lever to control the depth of induced magnetization *i.e.*, controlling the effective thickness of the magnetic layer. The observed deviations suggest that observations of ion-induced disorder may help refining existing simulation tools employed for ion-irradiation experiments in general.

Similarly, direct measurements of the lateral distribution of the magnetic flux density can prove valuable for predictive modeling of ion-induced magnetization. In the present case of ferromagnetic stripes lateral ion-scattering can imply that the non-ferromagnetic spacer regions are in fact narrower than the intended 100 nm spacer widths, due to blurring of the edges of the ferromagnetic nanostructures. Furthermore, the ferromagnetic-edge region shown in Fig. 4e is expected to possess a transient magnetization, over a lateral distance of 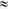
20 nm^8^.

We attempt to estimate the effect of lateral ion-scattering on the induced magnetization by comparing the flux density distribution around an ideal sharp ferromagnetic edge, with the flux density of a blurry edge considering lateral scattering. The effect of lateral scattering on the cross-sectional magnetization profile can be approximated using an s-shaped distribution:


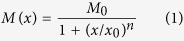


**Figure 6:**
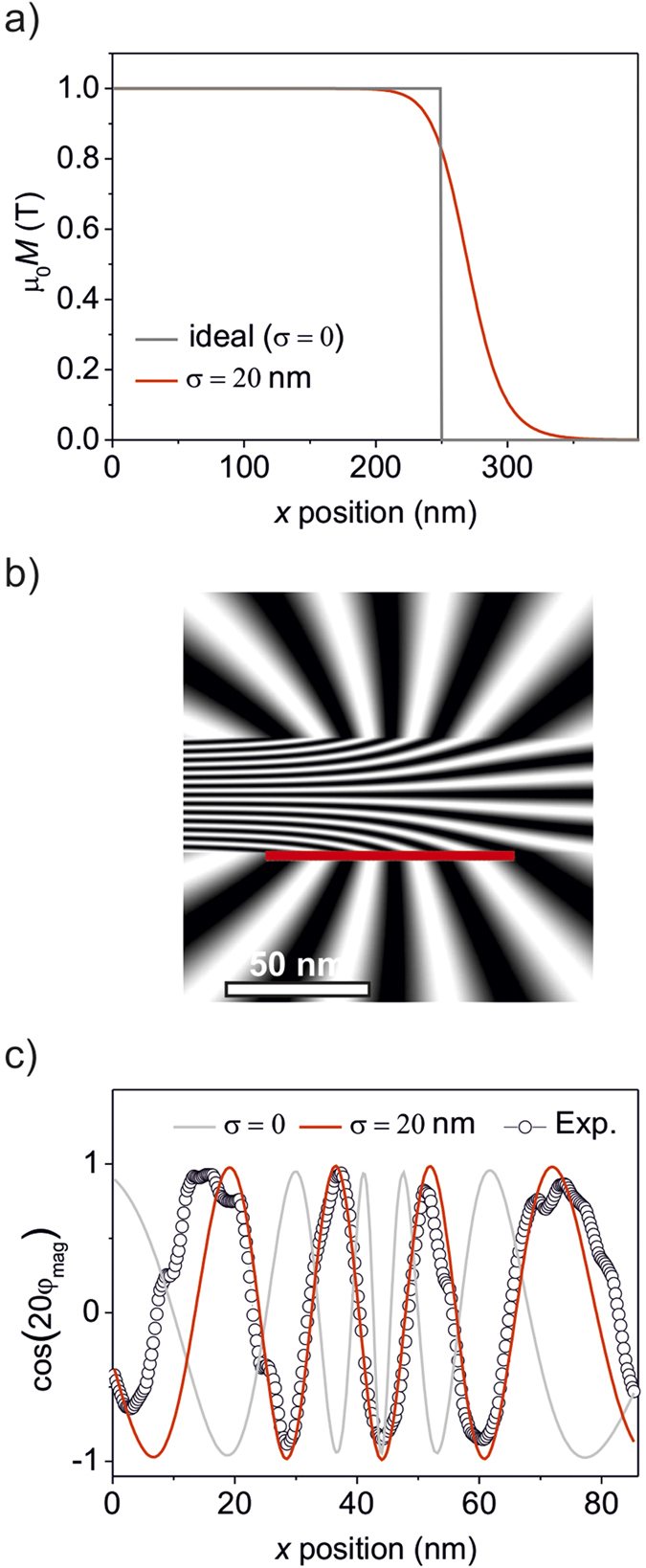
Simulation of the flux lines at a magnetic edge. (**a**) The ideal magnetization profile (grey) at the edge of a patterned ferromagnetic stripe, and the profile considering a lateral scattering 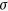 of 20 nm (red). (**b**) Simulation of the ferromagnetic edge shown in a) for the case of lateral ion-scattering. (**c**) Flux-density variation along the solid line in (**b**) where 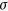 = 20 nm, the corresponding variation for 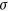 = 0 and the experimentally observed flux-density variation are plotted along this line.

*M*_0_ denotes the maximum magnetization (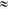
1 T), *x*_0_ is the distance of the edge from the center of the stripe and *n* is a fitting term that sets the steepness of the decay of magnetization at the edge. An ideal stripe ferromagnetic-edge is modelled by setting *x*_0_ = 250 nm and *n* → ∞, and is shown by the grey line in Fig. 6a. Setting *n* to finite values leads to a blurring of the ferromagnetic-edge, while values of *x*_0_ > 250 nm increase the effective width of the stripe, simulating possible effects of lateral scattering. The red line in Fig. 6a shows the magnetization profile for *x*_0_ = 270 nm, and *n* = 20. Note that at a position *x* = *x*_0_, *M* decreases to 0.5 *M*_0_. We define a lateral scattering term, 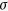, as the distance between the ideal edge, and the position where *M* = 0.5 *M*_0_. For the case considered in Fig. 6a, *x*_0_ is 270 nm giving a lateral scattering term 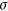 of 20 nm.

For the above calculations we considered stripes with dimensions of 500 nm × 40 nm × 76 nm, separated by 100 nm spacings. Milling of the lamella-like cross-section may have resulted in the exposure of its faces to Ga^+^ ions, thereby inducing a residual magnetization. Artefacts due to the magnetization induced during lamella preparation have been considered by assuming the existence of continuous of 5 nm thick magnetized layers on both faces of the lamella, leaving an undisturbed lamella thickness of 66 nm.

The above assumptions were applied to both the case of an ideal ferromagnetic stripe structure, and the one including the effect of lateral ion-scattering. The flux density distribution as seen by the magnetic phase shift for any arbitrary magnetic structure can be calculated by applying Maxwell’s equations (see e.g. ref. 23). In the present case Maxwell’s equations were applied to a set of two ferromagnetic stripes of the dimensions mentioned above (see Supplementary Information). In the first case the stripes possessed ideal edges with 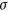 = 0, and in the second case, 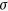 was set to 20 nm. The calculations were analytical for 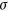 = 0, and numerical for 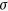 = 20 nm (see Supplementary Information) and yielded the spatial flux density distribution. In order to render the flux density as observed in the TEM instrument, we apply the same procedure as for the experiment, of integrating the magnetic flux density along the beam axis for every pixel in the image plane. Figure 6b shows the calculated flux density around the edge of a ferromagnetic stripe, setting 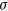 = 20 nm. The simulation result closely matches the experimentally observed flux density around the ferromagnetic edge, shown in Fig. 4e. Note the sharp bends in the flux lines that occur as they exit the continuous film and emerge at the interfaces. The positions of these exit points were found to depend sensitively on the magnetization distribution at the edge.

To illustrate the sensitivity of the positions of the exit points on the magnetization profile, Fig. 6c plots the variation of cos(20φ_mag_) along a line that runs horizontally along the film interface, in the vicinity of the ferromagnetic edge (solid red line in Fig. 6b). The peaks observed in Fig. 6c correspond to the location where a flux line bends sharply and exits the film. Figure 6c shows the cos(20φ_mag_) distribution along the interface for 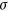 = 0 and 20 nm, and for the experimentally observed values extracted from Fig. 4e.

The clear differences in the positions of the exit points of the flux lines, for the two cases of 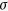 shown in Fig. 6c shows that the flux lines at the edge act as fingerprints of the magnetization profile. It is evident that the experimentally observed positions where the flux lines exit the film can only be fitted using a lateral scattering term of 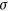 = 20 nm. The positions of the exit points are seen to be closer to each other for 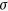 = 0, since the ideal magnetization profile causes much sharper bending of the flux lines at the interfaces. Lowering the steepness of the magnetization profile by setting a lateral scattering term of 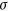 = 20 nm places the exit points almost exactly at the experimentally observed positions. This implies that for obtaining a given spacing between two magnetic objects, it is necessary to overcompensate the nominal spacing by 20 nm at each edge. The lateral scattering estimated using holography is larger than the 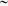
10 nm that were expected from semi-empirical modeling^8^.

The observations of this study are far more direct than the previous modeling work, and they help in achieving minimum possible non-ferromagnetic spacer distances. Narrow non-ferromagnetic spacers are of vital importance for spin-transport devices, wherein the spacing between two active ferromagnetic elements must be narrower than the spin-diffusion length. The width of the non-ferromagnetic spacing is also crucial in controlling the magneto-static coupling between neighbouring ferromagnetic elements at the nanoscale. Magnetically modulated materials consisting of arrays of ferromagnetic elements surrounded by narrow non-ferromagnetic spacers can show intriguing spin-wave resonances when pumped with microwave excitations. Such spin-wave or magnonic phenomena have been suggested as schemes for highly tuneable magneto-logic devices. Ion-induced ferromagnetism provides a convenient pathway to produce ferromagnetic elements with tailored magnetic coupling at the nanoscale for a variety of possible applications.

## Conclusions

Direct holographic observations have been made to map the depth- and lateral- distribution of *μ*_0_*M* where the magnetization, *M*, is induced by ions penetrating a system with disorder induced ferromagnetism. The depth distribution of *μ*_0_*M* can be controlled by varying the energy of the impinging ions. Homogenously magnetized thin films were formed by providing ions with sufficient energy to fully penetrate the film. Ferromagnetic nanostructures have been patterned using a nano-focussed ion-beam and the emergent flux line distribution observed using electron holography was used to fingerprint the magnetization profile in the vicinity of a ferromagnetic edge. The behavior of flux lines at the nanoscale can be modelled using Maxwell’s equations. This can open fascinating prospects in the field of nanomagnetism, whereby it may be possible to write nanomagnets of desired shapes onto materials that exhibit ion-induced ferromagnetism. Such structures may prove useful, for instance, as channels for spin-wave propagation and manipulation, as well as for lateral spin transport architectures. The flexibility with which the magnetic nanostructures can be written makes ion-induced ferromagnetism an ideal pathway for rapidly producing prototypes of novel magnetization configurations for use in future magnetic devices.

## Methods

### Films Growth

The Fe_60_Al_40_ thin films were deposited on a 150 nm thick SiO_2_ layer grown onto a silicon substrate. These films were prepared by magnetron sputtering and annealed for 1 hr at 773 K in vacuum. Annealing was necessary for the formation of the chemically ordered B2 phase, the existence of which was confirmed using X-ray diffraction, as well as Transmission Electron Microscopy. Unless otherwise stated, the film thickness was kept fixed at 40 nm. Disorder was created by concerted Ne^+^ ion irradiation at different kinetic energies ranging from 5–30 keV^8^. For electron holographic investigations, the samples were conventionally prepared in cross section using a dimpler and Ar-ion milling.

### Ion-irradiation using HIM

An Orion NanoFab Helium Ion Microscope (HIM) from Carl Zeiss AG has been used to pattern the nano magnets into the FeAl film. Helium Ion Microscopy utilizes a 0.5 nm wide beam of 30 keV Helium ions to visualize or structure samples at the nanoscale. Here, Neon has been used in the Gas field ion source^24^. In this way a probe size of 2 nm can be achieved. The patterning has been performed with the help of an FIBICS NPVE pattern generator. The beam conditions used are: 25 keV primary energy, 10 μm aperture with a resulting beam current of 0.8 pA at a spot control setting of 4. A pixel spacing of 1 nm has been used for the pattern generator. The beam has been scanned 6 times over each nano-magnet to achieve the target fluence of 6 Ne^+^-ions nm^–^^2^ and to ensure a uniform distribution of the damage events.

### TEM sample preparation

Classical TEM cross-sections of Ne^+^ broad-beam irradiated Fe_60_Al_40_ films glued together in face-to-face geometry using G2 epoxy glue (Gatan), were prepared by sawing (Wire Saw WS 22, IBS GmbH), grinding (MetaServ 250, Bühler), polishing (Minimet 1000, Bühler), dimpling (Dimple Grinder 656, Gatan) and final Ar^+^ ion milling (Precision Ion Polishing System PIPS 691, Gatan). To guarantee the reference wave passing through field-free space in the holography experiment, the classically prepared specimens were divided into two parts along the glue line with only one part being actually used for analysis.

TEM lamella preparation of the Ne^+^ion beam structured Fe_60_Al_40_ film was done by *in situ* lift-out using a Zeiss Crossbeam NVision 40 system. To protect the sensitive surface of the magnetically patterned film at the area of interest, a carbon cap layer was deposited beginning with electron beam assisted precursor decomposition and subsequently followed by Ga focused ion beam (FIB) assisted precursor decomposition. Afterwards, the TEM lamella was prepared using a 30 keV Ga FIB with adapted currents. The lamella was transferred to a 3 post copper lift-out grid (Omniprobe) using a Kleindiek micromanipulator. To minimize sidewall damage, Ga^+^ ions with energy limited to 5 keV were used for the final thinning of the TEM lamella to electron transparency.

### Electron holography

**Figure 7:**
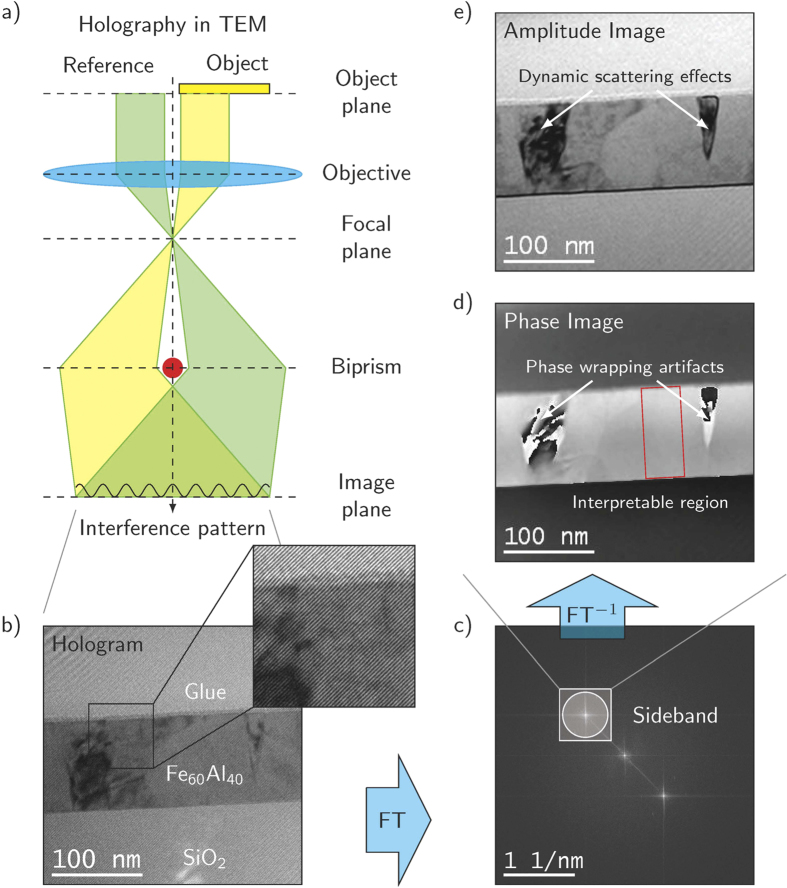
Acquisition and reconstruction of an electron hologram. (**a**) Set-up of Electron Holography in TEM. (**b**) Hologram of a 100 nm thick Fe_60_Al_40_ thin film grown on SiO_2_ substrate and covered with glue for TEM sample preparation. (**c**) Fourier spectrum of the hologram showing two sidebands and one center band. Fourier transform of the upper sideband low-pass filtered by a numerical aperture yields the image wave represented by the phase, (**d**) and amplitude image (**e**).

We apply Off-Axis Electron Holography^25^,^26^,^27^, in TEM to measure the formation of the magnetic fields within and around the magnetic nanostructures. This technique was successfully applied to measure magnetic fields with nanometer spatial resolution in the past^28^,^29^,^30^. By means of the Möllenstedt biprism^31^, an electron wave passing through the object is coherently superimposed with a reference wave passing through field-free space forming an interference fringe pattern -the hologram- at the detector plane (Fig. 7a,b). Electric and magnetic fields in the object shift the electron wave’s phase by elastic interaction leading to a local bending of interference fringes (inset in Fig. 7b). The reconstruction of amplitude and phase is performed by low-pass filtering of the sideband in the Fourier transform of the hologram (Fig. 7c) yielding amplitude and phase images (Fig. 7d,e) with a spatial resolution of about 5 nm.

*In the Wentzel-Kramers-Brillouin (WKB) approximation, the phase shift is given by:*


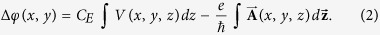


The first integral denotes the projection of the electric potential V and the second of the magnetic vector potential 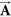 onto the beam path in z-direction. C_E_ is the interaction constant (7.29 V^−1^μm^−1^ for 200 keV electrons). To separate electric and magnetic phase shifts the method of *in-situ* magnetization reversal is applied^22^. We tilt the sample out of plane by α = ± 30° and saturate the sample by means of the magnetic field of the objective lens (2T). For both magnetization states we acquired series of holograms and reconstructed mean amplitude and phase images according to the procedure described in ref 32.

The wave series were aligned to cancel object and biprism drift as well as biprism charging by means of the Triebenberg software package^33^. The aligned wave series were averaged to reduce the noise level. Finally we obtain an averaged and normalized phase image for each magnetization state. The electric phase (φ_el_) image is given by the half sum and the magnetic phase (ϕ_mag_) image is given by the half difference of the two phase images, respectively. The projected in-plane component of the magnetic induction is determined by the gradient of the magnetic phase image and dividing the thickness determined from the electric phase shift. The latter step assumes a homogeneous magnetic induction along the beam axis within the sample. The mean in-plane components are given by:


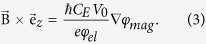


## Additional Information

**How to cite this article**: Röder, F. *et al.* Direct Depth- and Lateral- Imaging of Nanoscale Magnets Generated by Ion Impact. *Sci. Rep.* **5**, 16786; doi: 10.1038/srep16786 (2015).

## Supplementary Material

Supplementary Information

## Figures and Tables

**Figure 1:**
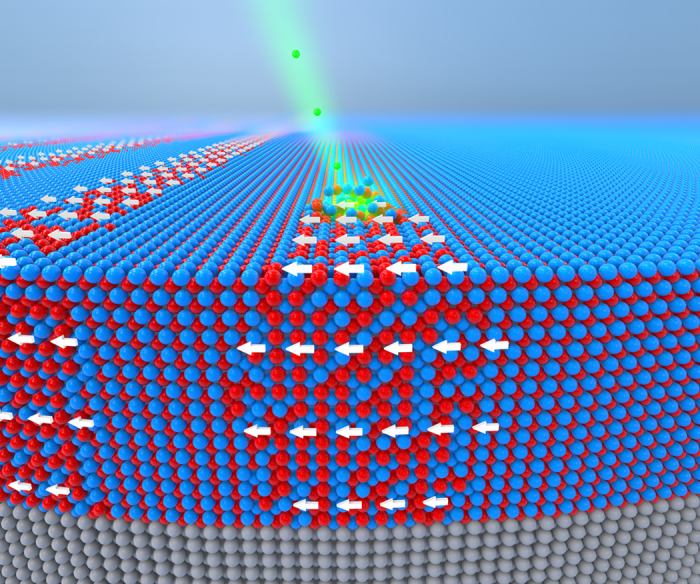
Direct magnetic writing using a nano-focussed ion-beam. A 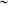 2 nm diameter Ne^+^ beam of an He-ion microscope can be used to generate confined atomic rearrangements at desired locations to induce magnetized regions.
