# Supplementary material for: Direct Depth- and Lateral- Imaging of Nanoscale Magnets Generated by Ion Impact
Source: Sci Rep. 2015 Nov 20;5:16786. doi: 10.1038/srep16786 (PMC4653643; doi:10.1038/srep16786)
Supplement: Supplementary Information [file srep16786-s1.pdf]

Supplementary Information

Direct Depth- and Lateral- Imaging of Nanoscale Magnets Generated by Ion Impact

Falk Röder,[†,*] Gregor Hlawacek,[‡] Sebastian Wintz,[‡] René Hübner,[‡] Lothar Bischoff,[‡] Hannes Lichte,[†] Kay Potzger,[‡] Jürgen Lindner,[‡] Jürgen Fassbender,[‡,§] and Rantej Bali[‡,*]

[†]Triebenberg Labor, Institut für Strukturphysik, Technische Universität Dresden, D-01062 Dresden, Germany

[‡]Helmholtz-Zentrum Dresden-Rossendorf, Institut für Ionenstrahlphysik und Materialforschung, Bautzner Landstraße 400, D-01328 Dresden, Germany

[§]Institut für Festkörperphysik, Technische Universität Dresden, Helmholtzstr. 10, D-01069 Dresden, Germany

1. Experimental details

We employ the FEI TECNAI F20 Cs-corrected transmission electron microscope to acquire the hologram series. Objective lens was switched-off during ensuring measurements in remanent state. For imaging we used the pseudo-Lorentz lens using the first transfer lens of the Cs corrector (CEOS company) providing a spatial resolution of 1 - 2 nm (diffraction lens at 82%). Sample magnetization and its reversal was achieved by tilting the sample out-of-plane by $\alpha = \pm 30°$ and applying the immersion field of the objective (2T). For $\alpha = 0°$, we acquired series of 20 holograms (2 - 3 s individual exposure times) according to the procedure in Ref. 32 (main article) for both remanent magnetization states. Additionally we acquire reference holograms without object to correct various artifacts.[S1] The wave series were reconstructed using a Butterworth filter of order 5 and a width realizing a spatial resolution of about 5 nm and normalized them by waves reconstructed from reference holograms. The reconstructed and normalized wave series were corrected with respect to object and biprism drift caused by instabilities during series acquisition. Object drift correction was achieved by minimizing the root mean square between subsequent amplitude images using non-linear fitting routines,[S2] realized with MATLAB™. Biprism drift leads to global phase offsets and biprism charging to phase wedges. Both were determined by fitting the phase in a vacuum region and subtracted from the reconstructed and normalized phase images. The averaging was conducted with respect to the wave to avoid artifacts due to phase wrappings. The noise discussed here corresponds to the estimated pixel-wise standard deviation of the mean values (see Ref. 32 main article).

The wave reconstruction and the (rough) correction of object and biprism drift were conducted by means of the Triebenberg-Software package (Ref. 33 of main article) written for DIGITALMICROGRAPH^{TM}. Phase unwrapping was conducted using the Goldstein-algorithm implemented by Ref. S3. For the two opposite magnetization directions, one obtains two mean phase images: The half sum returns the electric and the half difference the magnetic phase image. Here, displacements were corrected again as described above by means of matching the amplitude images. The vacuum regions were used again to determine and to remove phase-offset and tilt before determination of electric and magnetic phase images.

1.1 Mean Inner Potential (MIP) determination for $Fe_{60}Al_{40}$:

We determine the mean inner potential for $Fe_{60}Al_{40}$ by means of three different samples: Virgin as-grown, annealed at 500°C and irradiated with 10 keV ion beam energy. For these three samples we acquired and reconstructed hologram series as described above. The electric phase images show step-wise profiles due to different mean inner potentials of $Fe_{60}Al_{40}$, SiO_2 and silicon. We used the known value,[S4] for SiO_2 (10V) and varied the MIP for $Fe_{60}Al_{40}$ and Si to achieve a nearly continuous thickness profile as shown in Figure S1a. The resulting mean inner potentials are shown in Figure S1b. For the silicon we obtain (except the annealed case) values slightly below the known value,[S4] of 12 V, because of near-zone-axis orientation of the substrate. For $Fe_{60}Al_{40}$ we obtain an average value of 18(1)V, the error is due to uncertainties of the SiO_2 value, charging in the substrate and dynamical scattering in the thin film. Compared to the amplitude profiles (not shown here), we obtain a holographic mean free path of about 50(5) nm.

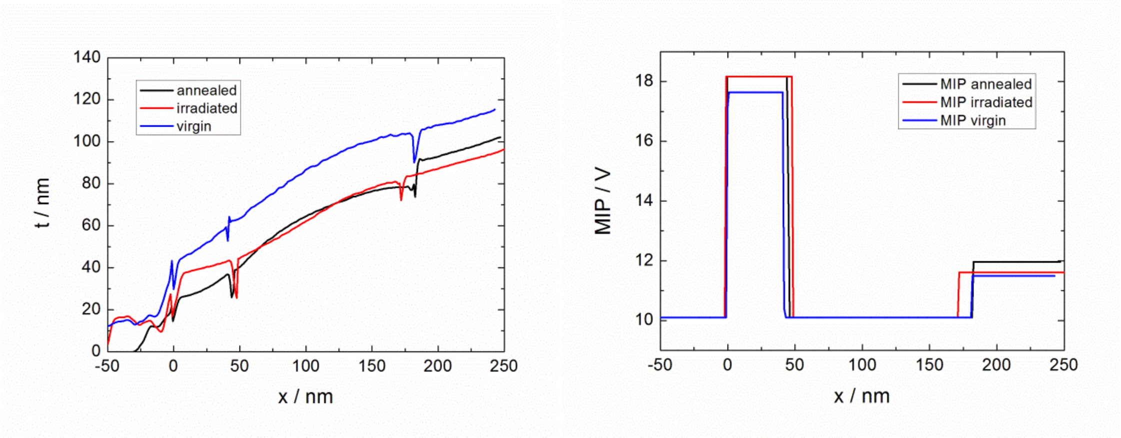

Figure S1: (Left) Thickness profiles obtained from phase images and **(Right)**, the Mean Inner Potential-profile. As reference value we have chosen 10 V for SiO_2 and 12 V for silicon in the annealed case.

1.2 Amplitude image of the stripe sample

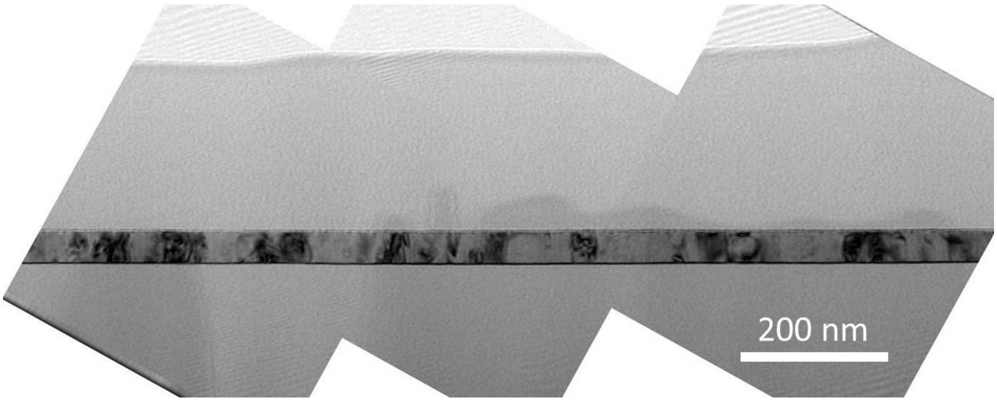

Figure S2: Amplitude image of the magnetic phase image shown in Figure 4b (main article).

The amplitude image of the magnetic phase image Figure 4b (main article) is presented in Figure S2 showing the polycrystalline nature of the thin film. It indicates that the spacer region Figure 4d (main article) is influenced by dynamic diffraction on nano-crystals, which makes a quantification of the flux density in the thin film difficult. The area of the right ending (Figure

4e, main article) exhibits no diffraction contrast, which allows a reliable comparison with simulation.

1.3 Holographic results for determining the magnetization profile

For magnetization profiling we observed, where possible, thicker sample positions to increase the signal of the inner part of the cross-sectional sample, which is not affected by sample preparation. Hologram series were acquired and reconstructed as described above. For the mean phase images, which are relevant for the magnetization profile determination, we obtain the following standard deviations of the mean values averaged along the thin film (Table S1).

The unwrapped electric phase shifts of the annealed and irradiated samples are depicted in Figure S3a. The vacuum region is on top, the SiO_2 substrate on bottom and the $Fe_{60}Al_{40}$ in between. In case of the 30 keV (100nm) sample, a large glue layer is observable in the vacuum region. The graining in the thin film visible in the electric phase image indicates the poly-crystal structure. This causes problems due to dynamical electron scattering, which is known to be strongly dependent on crystal orientation.[S5] Consequently, slight orientation mismatches due to mechanical hysteresis of the TEM goniometer induces phase differences showing up as local artifacts in the electric and magnetic phase images Figure S3a and b. That limits the size of the interpretable area of the thin film. Therefore we do only interpret quantitatively along certain profiles (horizontally averaged, see Table S2) indicated by vertical colored arrows. Figure S3c shows the corresponding step-like profiles due to different mean inner potentials. The ratios between the phase shifts of the thin film and the substrate for the 5 keV, 20 keV and 30 keV cases correspond well to the MIP ratio, while thicker samples (annealed, 10keV) show strong deviations. This is because of finite spatial resolution, which prohibits a correct sampling of the

very steep phase gradients at the interfaces. This facilitates phase unwrapping only partially, which is crucial for the thickness determination of the thicker sample positions (annealed and 10 keV). Especially 2π phase offset multiples are missing, which we had to figure out by comparison with the mean amplitude using the holographic mean free path of 50(5) nm (Table S2). This is important for correctly retrieving the thickness profile from the electric phase images (mean values summarized in Table S2). Finally we determine the thickness profile by linearly fitting the electric phase shift the region of the thin film and using the measured MIP of about 18(1) V as determined above.

Table S1: Standard deviations of the mean phase values in the thin film before and after magnetization reversal. The results are summarized in Figure S3.

**Magnetization**	**annealed**	**5 keV**	**10 keV**	**20 keV**	**30 keV (40nm)**	**30 keV (100nm)**
- 1 T	0.047(4)	0.051(1)	0.048(2)	0.045(8)	0.019(4)	0.035(3)
+ 1 T	0.047(3)	0.044(1)	0.055(5)	0.05(1)	0.019(4)	0.059(8)

Table S2: Mean thickness values retrieved from the profiles in Figure S3. Missing phase offsets were identified by comparing electric phase shift and amplitude images.

	**Annealed**	**5 keV**	**10 keV**	**20 keV**	**30 keV**	**30 keV (100nm)**
**Phase offset ϕ_0**	+ 6π	0	+ 6π	0	0	0
**Mean thickness [nm]**	230(20)	58(5)	200(20)	100(10)	75(5)	54(5)
**Horizontal averaging width [nm]**	110	33	22	220	220	22

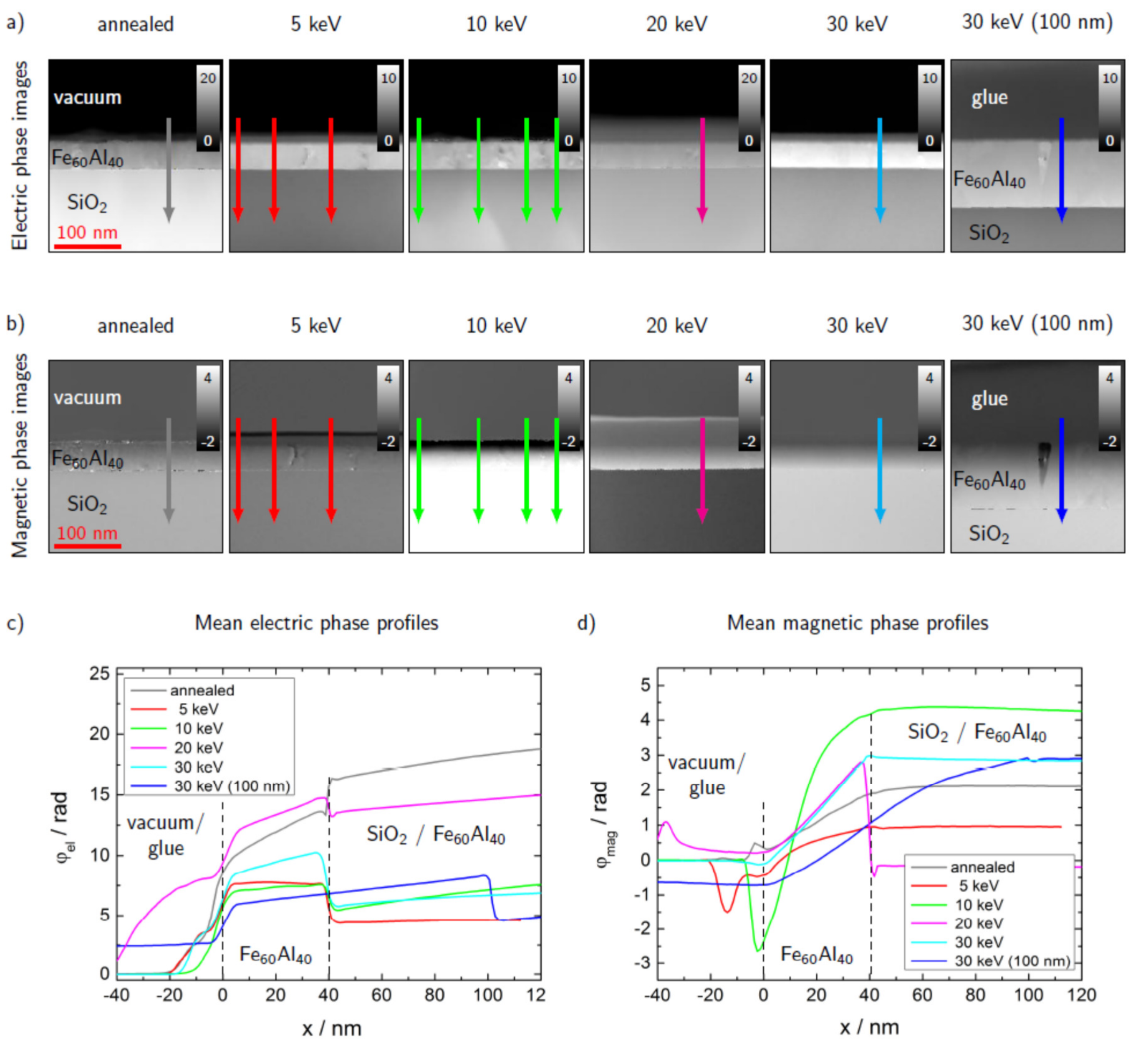

Figure S3: **(a)** Electric phase images determined by the sum of the reconstructed and phase unwrapped mean phase images before and after magnetization reversal. Arrows indicate the horizontally averaged line scans (see Table S2) of the data used for further analysis. Corresponding profiles are shown in **(c)** averaged over individual line profiles depicted in **(a)**. **(b)** Magnetic phase images are shown as difference image. **(d)** Corresponding profiles of the magnetic phase images are averaged in the same way as in **(c)**.

Another influence is given by contamination during hologram series acquisition, which had a crucial impact on the 30 keV (100nm) sample, which can be seen by the difference of the measured phase noise for the acquisitions before and after magnetization reversal, while the other samples show rather reproducible values. In the mean phase images we see differences of about 1 rad in the non-magnetic part indicating a growing of contamination after magnetization

reversal acting as magnetic dead layers on top and bottom of the sample. To correct for that we subtracted in this case 0.5 rad from the electric phase image before determining the thickness profile without contamination dead layers.

The corresponding magnetic phase images are shown in Figure S3b and their profiles in d. At the interfaces we observe artefacts due to phase unwrapping and image alignment mismatches. However these artifacts do not affect the interpretation of the magnetic phase images within $Fe_{60}Al_{40}$. The annealed sample exhibits a rather homogeneous slope in the thin film due to a finite magnetization, which is unexpected. We attribute this effect to disorder induced magnetism located on top and bottom of the sample due to TEM sample preparation. The irradiated samples exhibit inhomogeneous slopes due to spatially varying flux densities given by the different penetration depths of the irradiating ions. Correct interpretation in terms of local magnetic fields requires the local thickness profile extracted from the electric phase shifts (Figure S3c), by fitting linear function in the thin film region. Finally we find the distributions of the mean in-plane flux density in the thin film by means of Equation 3 as represented in Figure S4.

The case of 20 keV and 30 keV for the 40 nm thin film shows nearly the same phase gradient, whereas the electric phase strongly differs (magenta and cyan curves in Figure S3c and d). This presumably indicates the presence of non-ferromagnetic capping layers especially in the case of the 20 keV sample, which additionally shifts the electric phase but not the magnetic phase. As a consequence the thickness will be overestimated and the resulting B-field underestimated as shown in Figure S4.

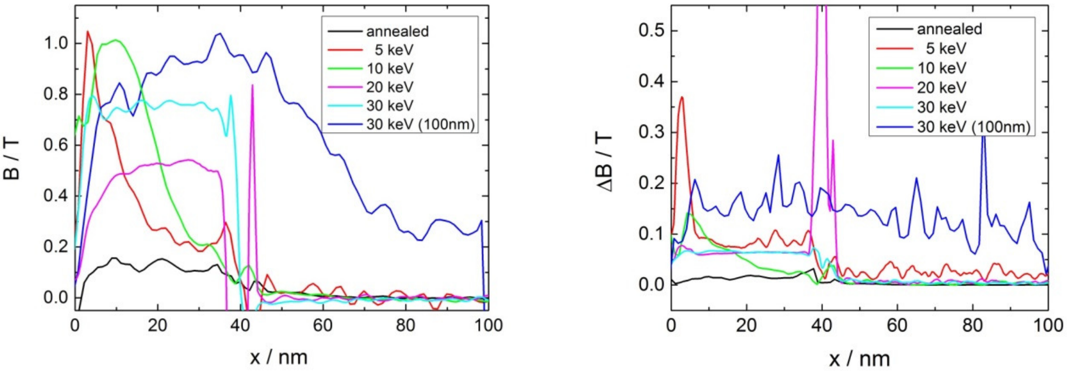

Figure S4: (Left) Mean in-plane flux density parallel to the surface using the experimental data of Figure S3 and Equation 3. Magnetization profiles depending on ion beam penetration depth are clearly visible although a non-vanishing background and magnetization in the annealed sample (ordered) is observable. Deviation at the interfaces, i.e. at 0, 40 nm and 100 nm are attributed to artifacts due alignment mismatches and phase wrappings at the steep edges in the phase images. **(Right)** Absolute error estimated of the reconstructed error as a consequence of the phase measurement and MIP determination (Error estimation).

2. Error estimation

According to Equation 3 we determine the relative error of the B-field by:

$$\left|\frac{\Delta B}{B}\right| = \left|\frac{\Delta\varphi_{el}}{\varphi_{el}}\right| + \left|\frac{\Delta\partial\varphi_{mag}}{\partial\varphi_{mag}}\right| + \left|\frac{\Delta V_{MIP}}{V_{MIP}}\right|$$

Error of the electric and magnetic phase shift

$$\varphi_{el} = \frac{\varphi_+ + \varphi_-}{2} \quad \text{and} \quad \varphi_{mag} = \frac{\varphi_+ - \varphi_-}{2}$$

We assume that the errors of the two independent measurements for φ_+ and φ_- are uncorrelated. Therefore it suffices to use the square sum of the variances $\Delta\varphi_+{}^2$ and $\Delta\varphi_-{}^2$ of the measured phase shifts:

$$\Delta\varphi_{el} = \Delta\varphi_{mag} = \frac{\sqrt{\Delta\varphi_+{}^2 + \Delta\varphi_-{}^2}}{2}$$

Note that the case of fully correlated errors yields $\Delta\varphi_{mag} = 0$. The error of the gradient determined from the noisy magnetic phase shift depends on the correlation between adjacent pixels. This correlation emerges from the Point Spread Function which determines the spatial resolution in the reconstructed phase image, which we assume to be Gaussian like:

$$PSF(r) = \frac{1}{2\pi\delta^2} e^{-\frac{r^2}{2\delta^2}}$$

For a spatial resolution of 5 nm we assume a FWHM = 10 nm, which implies a standard deviation of the Gaussian distribution of about $\delta = 4.26$ nm. The blurred phase image is given by the original phase shift φ_0 convoluted with the Point Spread Function. From this relation we infer the correlation coefficient by:

$$\Gamma(r, r') = \frac{cov(\varphi_{mag}(r), \varphi_{mag}(r'))}{\sqrt{cov(\varphi_{mag}(r), \varphi_{mag}(r))^2 cov(\varphi_{mag}(r'), \varphi_{mag}(r'))^2}}$$

We assume that the magnetic phase shift is much weaker changing than the PSF. That is, for the covariance we obtain the following approximation:

$$cov\big(\varphi(r), \varphi(r')\big) = \int PSF(r - r'')\varphi_0(r'')PSF\,(r' - r'')d^2r''$$

$$\approx \varphi_0(r) \int PSF(r - r'')PSF\,(r' - r'')d^2r''$$

$$\approx \varphi_0(r) \frac{1}{4\pi^2\delta^4} e^{-\frac{(r-r')^2}{4\delta^2}}.$$

For neighbored pixels the correlation factor yields:

$$\Gamma(r, r') \approx e^{-\frac{(r-r')^2}{4\delta^2}}$$

We evaluated the gradient pixel wise with a calibration of about Δr =1.1 nm per pixel. In this case the correlation coefficient for a spatial resolution of about 5 nm yields:

$$\Gamma(\Delta r) \approx e^{-\frac{(\Delta r)^2}{4\delta^2}} = 0.984$$

We determine the error of the magnetic phase gradient by:

$$\Delta\partial\varphi_{mag}(r) \approx \frac{\Delta\varphi_{mag}(r)}{\Delta r}\sqrt{2\big(1 - \Gamma(\Delta r)\big)} \approx 0.2\ \frac{\Delta\varphi_{mag}(r)}{\Delta r}$$

The relative error for the determination of the mean inner potential is given by:

$$\left|\frac{\Delta V_{MIP}}{V_{MIP}}\right| = \frac{1}{18}.$$

3. Effect of sample preparation induced disorder

TEM sample preparation damages the sample on top and bottom, which is crucial for the magnetic investigation of $Fe_{60}Al_{40}$, since disorder will end up with artificial ferromagnetic layers. The phase shift of the electron wave projects the in-plane flux density in beam direction. Consequently these artificial layers will contribute as background to the reconstructed phase images (Figure S3). Due to the projection property, it is justified to subtract the background. However, the thickness t determined from the electric phase shift will underestimate the mean flux density, which requires figuring out the effective thickness t_{eff} of the undisturbed magnetic region.

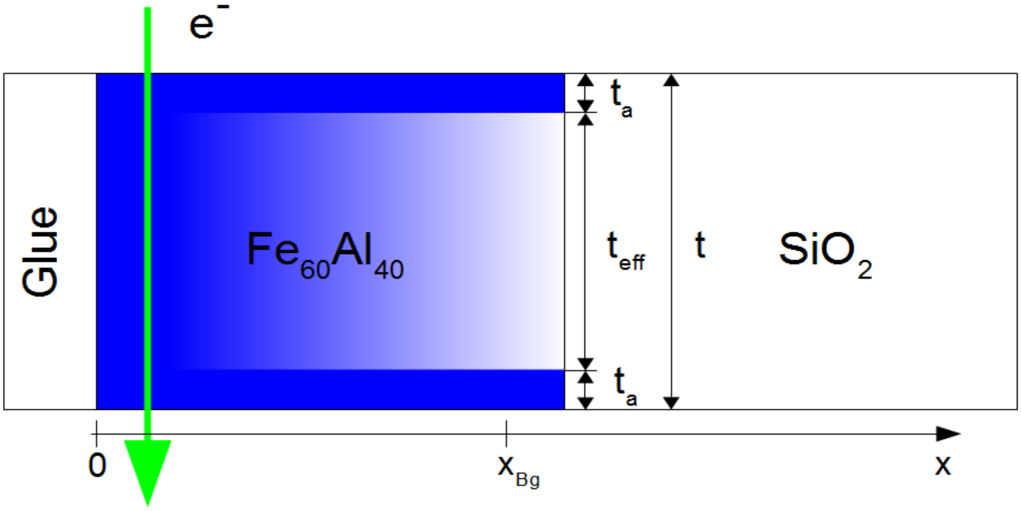

Figure S4 Cross-sectional scheme of the $Fe_{60}Al_{40}$ thin film as prepared for TEM investigations by Ar^{+} ion milling or FIB. The transmitted beam in TEM is shown by the green arrow. Ion irradiation was conducted from the left. Disorder induced ferromagnetic regions are colored in blue, non-ferromagnetic regions in white. Sample preparation induces artificial magnetized layer on top and bottom of the sample with thickness t_a.

This motivated to model the background in the following way: We assume that the magnetic phase gradient composes of:

$$\frac{\hbar}{e}\frac{d\varphi}{dx} = \int_0^{t_a} B_a(x,z)dz + \int_{t_a}^{t-t_a} B_m(x,z)dz + \int_{t-t_a}^{t} B_a(x,z)dz.$$

The mean magnetic field is determined by normalizing with the total thickness t of the sample:

$$< B(x) >= \frac{1}{t}\frac{\hbar}{e}\frac{d\varphi}{dx}.$$

To determine the background contribution we chose the region close to the $Fe_{60}Al_{40}/SiO_2$ for $x = x_{Bg}$ (Figure S4):

$$< B_{Bg} >= \frac{1}{t}\left(\int_0^{t_a} B_a(x_{Bg},z)dz + \int_{t-t_a}^{t} B_a(x_{Bg},z)dz\right) = \frac{2t_a}{t}B_a.$$

Here, we further assume that the damaged areas are homogeneously magnetized and yield a flux density of B_a = 1.0 T in remanence, which defines the thickness of the artificial layers. The subtraction of the mean background field yields:

$$< B(x) > - < B_{Bg} > = \frac{1}{t}\int_{t_a}^{t-t_a} B_m(x,z)dz = \left(1 - \frac{2t_a}{t}\right) B_m(x).$$

For the last step we assumed that the B-field B_m in the undisturbed region is homogeneous in beam direction (z). We finally substitute t_a by the expression above and obtain the flux density B_m within the undisturbed region:

$$B_m(x) = \frac{< B(x) > - < B_{Bg} >}{1 - \frac{< B_{Bg} >}{B_a}}.$$

From the results in Figure S3 we extract the background field $<B_{Bg}>$ by the minimal value within the $Fe_{60}Al_{40}$ thin film.

Table S3 Data for background removal.

**Sample**	**$<B_{Bg}>$ \ T**	**$<t>$/nm**	**t_a/ nm**
Annealed	0.1	230	12
5 keV	0.2	58	6
10 keV	0.05	200	5
30 keV	0.25	54	7

Using the data of Table S3, we obtain the final profiles of the magnetic field as shown in Figure 5 (main article).

4. Magnetostatic calculation of the phase image

For the phase shift given by a homogeneously magnetized bar of length L, width b and height h as depicted in Figure S5.

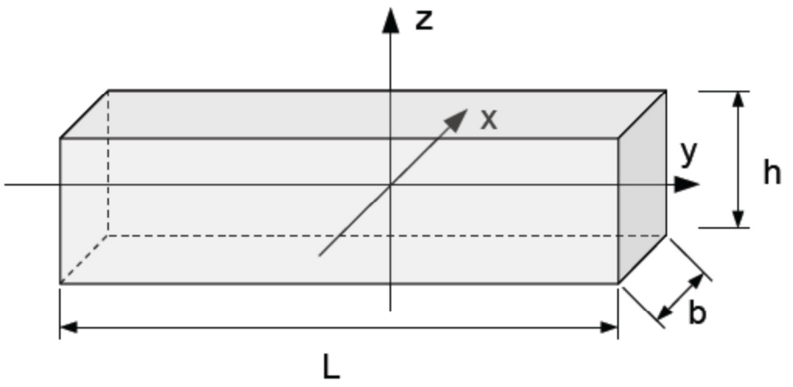

Figure S5: Geometry of a bar magnetized in the long-axis direction.

We need to solve first the magnetostatic boundary problem yielding the magneto-static potential ψ for the H-field:

$$\psi(x,y,z)=\frac{1}{4\pi}\int_{-\frac{b}{2}}^{+\frac{b}{2}}\mathrm{d}x'\int_{-\frac{h}{2}}^{+\frac{h}{2}}\mathrm{d}z'\left[\frac{M}{\sqrt{(x-x')^2+(y-\frac{L}{2})^2+(z-z')^2}}-\frac{M}{\sqrt{(x-x')^2+(y+\frac{L}{2})^2+(z-z')^2}}\right]$$

Second we assume the biprism to be aligned parallel to the long axis and the position x_0 as a field-free reference point. The phase shift due to the H-field with respect to this point is given by

the following integral of the y-derivative of magnetostatic potential (a consequence of Eq. 3 in the main article).

$$\Delta\varphi_{Hy}(x,y) = -\int_{x_0}^{x} \mathrm{d}x' \int_{-\infty}^{+\infty} \mathrm{d}z' \frac{\partial}{\partial y}\psi(x',y,z')$$

The analytic solution of this integral, which is equivalent to the solution found in Ref. 23 (main article), yields:

$$\begin{aligned}
\Delta\varphi_{Hy}(x,y) &= -\frac{M}{4\pi}\mu_0\frac{e}{\hbar}\int_{x_0}^{x}\mathrm{d}x' \int_{-\infty}^{\infty}\mathrm{d}x''\, h\frac{\partial}{\partial y}\ln\left[\frac{(x'-x'')^2+(y-\frac{L}{2})^2}{(x'-x'')^2+(y+\frac{L}{2})^2}\right] \\
&= -\frac{M}{4\pi}\mu_0\frac{e}{\hbar}\cdot 2h \\
&\quad \left[\frac{y+\frac{L}{2}}{|y+\frac{L}{2}|}\left\{\left(x-\frac{b}{2}\right)\arctan\left(\frac{x-\frac{b}{2}}{|y+\frac{L}{2}|}\right)-\left(x_0-\frac{b}{2}\right)\arctan\left(\frac{x_0-\frac{b}{2}}{|y+\frac{L}{2}|}\right)\right.\right. \\
&\quad -\left(x+\frac{b}{2}\right)\arctan\left(\frac{x+\frac{b}{2}}{|y+\frac{L}{2}|}\right)+\left(x_0+\frac{b}{2}\right)\arctan\left(\frac{x_0+\frac{b}{2}}{|y+\frac{L}{2}|}\right) \\
&\quad \left.-\frac{|y+\frac{L}{2}|}{2}\ln\left[\frac{(x-\frac{b}{2})^2+(y+\frac{L}{2})^2}{(x_0-\frac{b}{2})^2+(y+\frac{L}{2})^2}\right]+\frac{|y+\frac{L}{2}|}{2}\ln\left[\frac{(x+\frac{b}{2})^2+(y+\frac{L}{2})^2}{(x_0+\frac{b}{2})^2+(y+\frac{L}{2})^2}\right]\right\} \\
&\quad -\frac{y-\frac{L}{2}}{|y-\frac{L}{2}|}\left\{\left(x-\frac{b}{2}\right)\arctan\left(\frac{x-\frac{b}{2}}{|y-\frac{L}{2}|}\right)-\left(x_0-\frac{b}{2}\right)\arctan\left(\frac{x_0-\frac{b}{2}}{|y-\frac{L}{2}|}\right)\right. \\
&\quad -\left(x+\frac{b}{2}\right)\arctan\left(\frac{x+\frac{b}{2}}{|y-\frac{L}{2}|}\right)+\left(x_0+\frac{b}{2}\right)\arctan\left(\frac{x_0+\frac{b}{2}}{|y-\frac{L}{2}|}\right) \\
&\quad \left.\left.-\frac{|y-\frac{L}{2}|}{2}\ln\left[\frac{(x-\frac{b}{2})^2+(y-\frac{L}{2})^2}{(x_0-\frac{b}{2})^2+(y-\frac{L}{2})^2}\right]+\frac{|y-\frac{L}{2}|}{2}\ln\left[\frac{(x+\frac{b}{2})^2+(y-\frac{L}{2})^2}{(x_0+\frac{b}{2})^2+(y-\frac{L}{2})^2}\right]\right\}\right]
\end{aligned}$$

This solution renders the phase shift of the external stray field. The total phase shift of the sample requires adding the phase shift for the magnetization contribution by:

$$\Delta\varphi_B(x,y) = \Delta\varphi_H(x,y) + \frac{e}{\hbar}\mu_0 M_{Bar}(x,y)hx$$

Note the $M_{Bar}(x,y)$ is equal to M within the bar and zero outside.

Based on this analytic solution of the magnetic phase shift of a homogeneously magnetized bar we incorporate the effect of lateral ion-scattering by decomposing the blurred magnetization profile (Figure 4c, main article, red line) into a set of bars of incremental magnetization and varying length. The sum over all bars renders the magnetization profile (Figure 4c, main article). For each sub-bar we apply the above analytic solution for the phase image and sum all results to obtain the final image (due to linearity of the field equations).

References

S1. H. Lichte, D. Geiger, A. Harscher, E. Heindl, M. Lehmann, D. Malamidis, A. Orchowski, and W.-D. Rau, *Ultramicrosc.*, **1996**, *64*, 67.

S2. T. Niermann, M. Lehmann, *Micron,* **2014**, 63, 28.

S3. P. Perkes, Phase Unwrapping Plug-in for Digital Micrograph, Arizona State University, AZ **2002**.

S4. Y. C. Wang, T. M. Chou, M. Libera, T.F. Kelly, *Appl. Phys. Lett.* **1997,** 70, 1296.

S5. Lubk, D. Wolf, and H. Lichte, *Ultramicrosc.*, **2010**, 110, 438.
